# NMR Spectroscopy for Metabolomics Research

**DOI:** 10.3390/metabo9070123

**Published:** 2019-06-27

**Authors:** Abdul-Hamid Emwas, Raja Roy, Ryan T. McKay, Leonardo Tenori, Edoardo Saccenti, G. A. Nagana Gowda, Daniel Raftery, Fatimah Alahmari, Lukasz Jaremko, Mariusz Jaremko, David S. Wishart

**Affiliations:** 1 Core Labs, King Abdullah University of Science and Technology (KAUST), Thuwal 23955-6900, Saudi Arabia; 2 Centre of Biomedical Research, Formerly, Centre of Biomedical Magnetic Resonance, Sanjay Gandhi Post-Graduate Institute of Medical Sciences Campus, Uttar Pradesh 226014, India; 3 Department of Chemistry, University of Alberta, Edmonton, AB T6G 2W2, Canada; 4 Department of Experimental and Clinical Medicine, University of Florence, Largo Brambilla 3, 50134 Florence, Italy; 5 Laboratory of Systems and Synthetic Biology Wageningen University & Research, Stippeneng 4, 6708 WE Wageningen, The Netherlands; 6 Northwest Metabolomics Research Center, Department of Anesthesiology and Pain Medicine, University of Washington, 850 Republican St., Seattle, WA 98109, USA; 7 Fred Hutchinson Cancer Research Center, 1100 Fairview Avenue, Seattle, WA 98109, USA; 8 Department of NanoMedicine Research, Institute for Research and Medical Consultations (IRMC), Imam Abdulrahman bin Faisal University, Dammam 31441, Saudi Arabia; 9 Division of Biological and Environmental Sciences and Engineering (BESE), King Abdullah University of Science and Technology (KAUST), Thuwal 23955-6900, Saudi Arabia; 10 Department of Biological Sciences, University of Alberta, Edmonton, AB T6G 2E8, Canada

**Keywords:** metabolomics, NMR, MS, analytical platform, GC-MS, LC-MS, sensitivity, resolution

## Abstract

Over the past two decades, nuclear magnetic resonance (NMR) has emerged as one of the three principal analytical techniques used in metabolomics (the other two being gas chromatography coupled to mass spectrometry (GC-MS) and liquid chromatography coupled with single-stage mass spectrometry (LC-MS)). The relative ease of sample preparation, the ability to quantify metabolite levels, the high level of experimental reproducibility, and the inherently nondestructive nature of NMR spectroscopy have made it the preferred platform for long-term or large-scale clinical metabolomic studies. These advantages, however, are often outweighed by the fact that most other analytical techniques, including both LC-MS and GC-MS, are inherently more sensitive than NMR, with lower limits of detection typically being 10 to 100 times better. This review is intended to introduce readers to the field of NMR-based metabolomics and to highlight both the advantages and disadvantages of NMR spectroscopy for metabolomic studies. It will also explore some of the unique strengths of NMR-based metabolomics, particularly with regard to isotope selection/detection, mixture deconvolution via 2D spectroscopy, automation, and the ability to noninvasively analyze native tissue specimens. Finally, this review will highlight a number of emerging NMR techniques and technologies that are being used to strengthen its utility and overcome its inherent limitations in metabolomic applications.

## 1. Introduction 

Similar to all other -omic sciences, metabolomics is a technology-driven discipline. It is constantly evolving and taking advantage of new developments in analytical chemistry, including analytical techniques, instrumentation, analytical software, statistical methods, or computational techniques to accelerate or improve data collection, data analysis, and data interpretation. The primary analytical technologies used in metabolomics include liquid chromatography coupled with single-stage mass spectrometry (LC-MS) [1,2,3] or tandem mass spectrometry (LC-MS/MS) [4,5,6,7,8,9], gas chromatography coupled to mass spectrometry (GC-MS) [1,2,10,11], high or ultrahigh performance liquid chromatography coupled to UV or fluorescent detection (HPLC/UPLC) [12,13,14,15,16,17,18,19], and nuclear magnetic resonance (NMR) spectroscopy [20,21,22,23,24,25,26,27]. Each analytical platform has its own advantages and disadvantages. The choice of the platform depends primarily on the focus of the study as well as on the nature of samples [28]. However, the selection of a given platform or platforms is also often determined by the cost, its accessibility, and the available expertise. It is important to remember that there is no single analytical platform that can perform a complete identification and quantification of all metabolites for a typical biological sample. As a result, the best metabolomic studies often employ multiple technology platforms.

**Figure 1:**
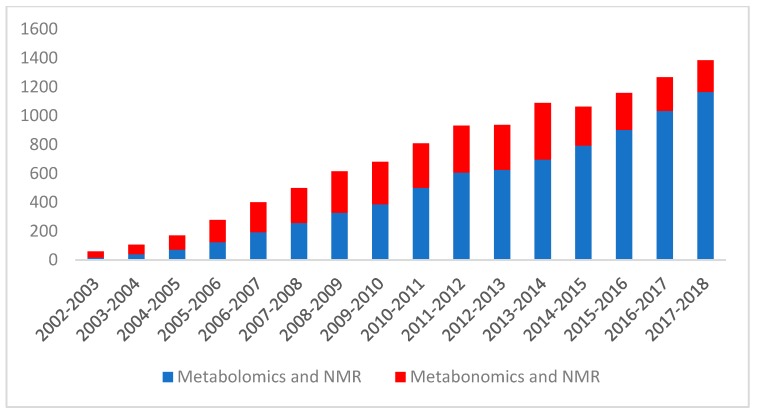
Increasing trend in the NMR-based metabolomics/metabonomics publications obtained using the keywords metabolomics and NMR (blue) or metabonomics and NMR (red) from the web of knowledge (http://apps.webofknowledge.com).

Today LC-MS, GC-MS, and NMR spectroscopy are the three most commonly used analytical methods in metabolomics. While LC-MS and GC-MS methods are becoming increasingly popular (accounting for more than 80% of published metabolomics studies to date), there is still considerable interest in using NMR-based methods for metabolomic studies. Figure 1 shows a plot of the steadily increasing number of NMR-based metabolomics/metabonomics papers for the past 15 years. This growth in the use of NMR for metabolomics reflects the fact that NMR has a number of unique advantages [29] over other metabolomic platforms such as LC-MS or GC-MS. In particular, NMR spectroscopy is nondestructive, nonbiased, easily quantifiable, requires little or no chromatographic separation, sample treatment, or chemical derivatization, and it permits the routine identification of novel compounds. Furthermore, NMR is highly automatable and exceptionally reproducible, making high-throughput [30], large-scale metabolomics studies much more feasible with NMR spectroscopy than with LC-MS or GC-MS. In addition to these strengths, NMR is particularly amenable to detecting and characterizing compounds that are less tractable to LC-MS analysis such as sugars, organic acids, alcohols, polyols, and other highly polar compounds. 

**Figure 2:**
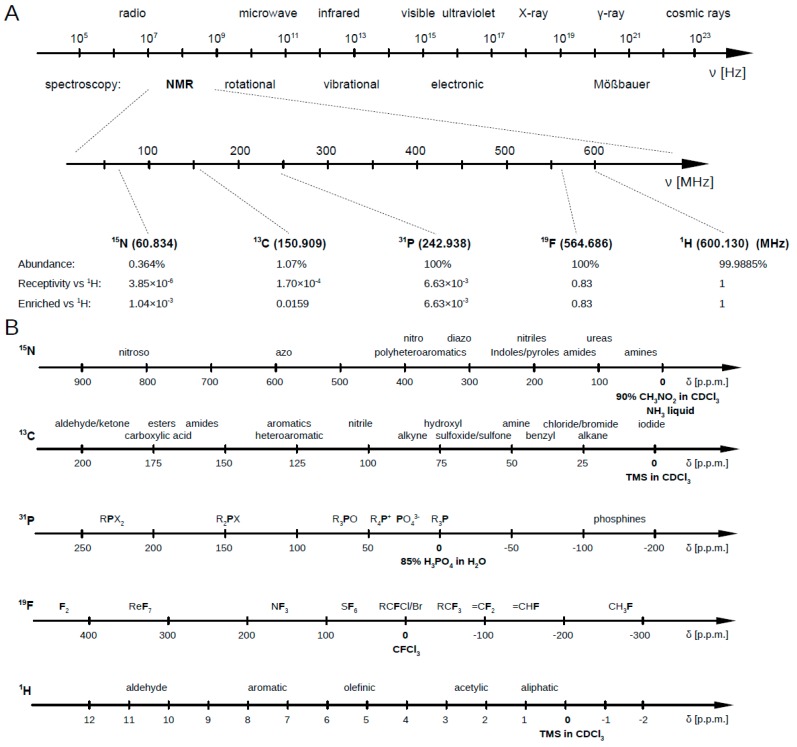
The electromagnetic nature of the NMR spectroscopy of the most common nuclei for metabolomics studies. (**A**) Frequency scale ranges and types of spectroscopies that correspond to them. The NMR frequency range for the most commonly used nuclei at 600 MHz proton frequency along with the natural abundances of the nuclei are also given. (**B**) Typical ppm ranges for the ^15^N, ^13^C, ^31^P, ^19^F, and ^1^H nuclei under different chemical environments.

Unlike most other metabolomic platforms, NMR is not restricted to biofluid or tissue extract analysis. It is well suited for studying intact tissues, organs, and other solid or semisolid samples through solid-state NMR (ssNMR) and magic-angle sample spinning (MAS-NMR) [31,32,33,34]. Moreover, researchers can record NMR spectra for multiple different nuclei (such as ^1^H, ^13^C, ^15^N, and ^31^P) either separately or simultaneously to study different metabolite classes (i.e., nitrogen-containing, phosphorous-containing, etc.). Furthermore, the correlation between two and even three different nuclei can be measured using multidimensional NMR methods (Figure 2). 

NMR also supports metabolite imaging and metabolic analysis of living samples through magnetic resonance spectroscopy (MRS) and magnetic resonance imaging (MRI) [35,36,37,38,39]. In contrast, LC-MS-based and GC-MS methods, because of their inherently destructive nature, cannot be used to analyse living samples. As a result, NMR is ideal for real-time metabolite profiling of living cells, and so real-time metabolic flux analysis can only be performed using NMR spectroscopy [40,41]. Additionally, NMR allows users to explore chemistry with much greater detail than any other method. It allows users to look at intact molecules at an atomic level and to see not just the ^1^H atoms but also many other kinds of atoms (^13^C, ^15^N) or biologically reactive groups, including phosphate atoms (^31^P) [42,43,44,45,46,47]. Furthermore, NMR spectroscopy can be used to assess unique classes of metabolites (especially protein-bound metabolites such as lipoprotein particles) and to measure certain inorganic metabolites or ions (metal ions and H+ ions via pH) that cannot be done via LC-MS or GC-MS [48,49].

However, NMR also has a number of disadvantages, with the most significant challenge for NMR being its lack of sensitivity. Compared to LC-MS and GC-MS, NMR spectroscopy is often 10 to 100 times less sensitive. This means that a typical NMR-based metabolomic study usually only returns information on 50–200 identified metabolites with concentrations >1 μM, while a typical LC-MS study can return information on 1000+ identified metabolites with concentrations of >10 to 100 nM. Table 1 summarizes the advantages and disadvantages of NMR spectroscopy compared to MS with particular focus on metabolomics applications. 

Over the past decade there have been a number of excellent reviews published on NMR and its applications in metabolomics [50,51,52,53]. However, most of these are now somewhat dated, while many others are focused on a particular aspect of NMR and metabolomics (i.e., quantitative NMR [50,54,55] or NMR methodology [56,57] or the future of NMR in metabolomics [56]). Our intent with this manuscript is to provide a more up-to-date perspective on NMR in metabolomics and to cover a number of areas not previously covered by other reviews. This review will not cover topics such as NMR sample preparation or NMR data processing; these subjects have recently been covered by a number of excellent and comprehensive reviews, some of which were previously written by members of our team [58,59,60]. Instead, this review will focus on NMR instrumentation, NMR techniques, and NMR software resources along with a critical assessment of available NMR spectroscopic techniques. Herein we will critically assess the strengths and weaknesses of available NMR spectroscopic techniques, technologies, and methodologies as they relate to metabolomics. Five main topics will be presented. The first will provide a short introduction to ^1^H NMR-based metabolomics followed by a critical assessment of the use of other types of nuclei (^13^C, ^15^N, and ^31^P) for NMR-based metabolomics, with a special focus on 1D NMR spectroscopy. The second topic will focus on 2D NMR spectroscopy for metabolomics and its potential advantages and disadvantages relative to 1D NMR. The third topic will discuss the availability and use of NMR databases and NMR software for metabolite identification and quantification. The fourth topic will critically assess a number of new methodological developments in NMR-based metabolomics with a focus on a) high-resolution magic-angle sample spinning (HRMAS) NMR for tissue metabolomics; b) rapid 2D acquisition methods for biofluid metabolomics; and c) sensitivity enhancements with hyperpolarization methods. The fifth topic will focus on new instrument developments that are impacting NMR-based metabolomics including the role that new magnets, new probes, and new low-volume NMR tubes are having on the field. This review will conclude with a discussion on the limitations of NMR-based metabolomics, some comments on the future of NMR-based metabolomics, and a few thoughts of where it needs to go to remain competitive in the fast-changing landscape of metabolomics. 

## 2. ^1^H NMR Spectroscopy for Metabolomics

**Figure 3:**
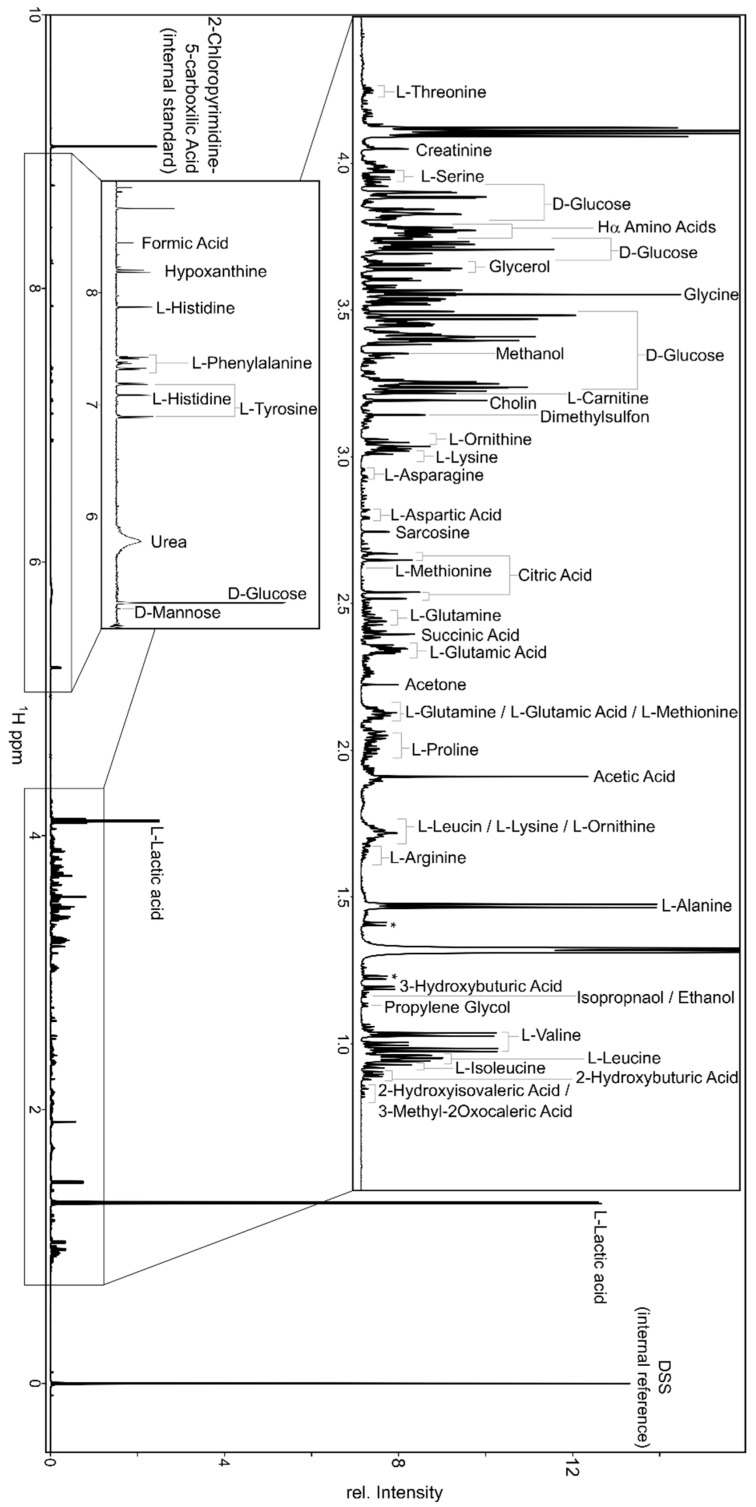
Annotated 1D ^1^H NMR spectrum collected of NIST SRM-1950 human serum (ultrafiltered with a 3 kDa MW cutoff filter) at 700 MHz. The NIST SRM-1950 sample is a pooled human serum sample collected from a large number of volunteers and distributed by the National Institute of Standards. The identified compounds are labeled above each of the corresponding peaks. The high lactate peak is due the fact that the sample had not been metabolically quenched by NIST during its preparation, leading to the conversion of glucose to lactate.

For this review it is assumed that the reader is at least modestly familiar with NMR spectroscopy and its general principles. If not, there are a number of excellent reviews that have been written over the past two decades that provide an excellent introduction to the principles and practice of NMR [28,61,62]. In this section we will focus primarily on the description of proton (^1^H) NMR spectroscopy (^1^H NMR), which is employed in the vast majority of NMR-based metabolomics studies. This is because ^1^H atoms are found in almost every organic compound and, therefore, almost every known metabolite. Figure 3 illustrates an example of a one-dimensional (1D) ^1^H NMR spectrum collected from a biofluid (human serum) that contains about 55 identifiable metabolites. As can be seen from this spectrum, there are hundreds of distinct signals arising for compounds that each contain one or more ^1^H atoms in their chemical structures. In addition to their ubiquity, ^1^H atoms exhibit, by far, the greatest NMR signal intensity (i.e., only ^19^F atoms are close in sensitivity), have very high (~99%) isotopic natural abundance, and exhibit remarkably narrow line widths (often <1 Hz). These narrow line widths are what give the spectrum in Figure 3 its exceptional resolution. 

One-dimensional ^1^H NMR spectra are particularly useful for metabolomic studies. This is because 1D ^1^H NMR, as a technique, is highly automatable, very reliable, and very fast. Indeed, 1D ^1^H NMR collection times for a single spectrum are often as short as a few minutes. With modern NMR equipment, NMR samples can be continuously loaded and removed with robotic sample exchangers that run for days or weeks at a time. Furthermore, the chemical information contained in a single 1D ^1^H NMR spectrum of a biofluid or tissue extract is often sufficient to identify and quantify 50–100 metabolites at a time [63,64]. This identification process is greatly aided by the fact that many reference ^1^H NMR spectra from hundreds of known metabolites have been compiled and stored in a number of public databases (see below). Additionally, many software tools exist to automatically or semi-automatically process and analyze 1D ^1^H NMR spectra (offline) within minutes or seconds after the spectra have been collected [65,66,67,68]. These software tools can both identify [68,69,70] and quantify [67,70] metabolites. Because of its advantages in speed and reproducibility, ^1^H NMR metabolomics has become the preferred technique for large-scale population studies where thousands or even tens of thousands of samples must be analyzed [71,72,73]. 

Compared to all other forms of NMR spectroscopy, simple 1D ^1^H NMR spectra are particularly useful for quantifying metabolites. This is because they are normally acquired under fully relaxed conditions and without polarization transfer techniques. Thus, ^1^H NMR signals acquired in this way provide a real representation of the distribution of proton nuclei within the molecules and the different concentration levels of the corresponding metabolites in a complex mixture. Of course, this assumes good instrument/magnet stability, consistent excitation pulse widths, appropriate experimental data acquisition intervals, and proper tune/match optimization. Also, quality controls have to be employed to maintain confidence in the spectrometer performance over the course of the study. Accurate quantitation is one of the hardest undertakings of NMR, but it is vital to the metabolomics data. 

Because almost all 1D ^1^H NMR spectra acquired for metabolomic studies are performed in water, solvent suppression is an important aspect of spectral acquisition that cannot be avoided. Depending on the nature of metabolites studied, different solvent suppression schemes or protocols may be used. For instance, if one is only interested in the non-exchanging protons in a sample, then the water suppression issue can be essentially avoided by the use of >99.9% deuterated solvents (D_2_O instead of H_2_O). In addition to solvent replacement methods, which often require lyophilization, there are also a variety of NMR pulse sequence techniques available for solvent suppression. Techniques used for effective and selective solvent suppression have been covered extensively by other reviews [74,75,76,77,78] and, therefore, will not be discussed in detail here.

While there are many efficient techniques to reduce the solvent’s ^1^H signal, most require careful preparation, exceptional patience or skill in their optimization, or are difficult to perform consistently for the inexperienced (or even modestly experienced) NMR spectroscopist. One method that is relatively simple to perform involves collecting the first increment of the 2D-^1^H,^1^H-nuclear Overhauser effect spectroscopy (NOESY) experiment. This method has been among the most often used, as it provides a reproducible and easy-to-implement experiment for recording 1D ^1^H spectra of biological samples with good water suppression [79]. As a result, this pulse sequence has become the predominant approach used by NMR researchers in metabolomics, although it has been shown that there can be complications in its use [80,81,82]. 

Because NMR-based metabolomic studies typically involve the collection of dozens to hundred (even thousands) of spectra, consistency across experiments is a key factor that must be considered when implementing solvent suppression. This is because errors in solvent suppression can have substantial, detectable effects on downstream data analysis. Solvent suppression is heavily influenced by parameter changes, especially those involving pulse sequence timings and power level settings. The single largest challenge is the unintentional effect of any solvent suppression technique on neighboring resonances around the water peak or in exchange with water. No technique or techniques (to our knowledge) have been developed that has effective and complete isolation of the solvent (water) resonance position. The power level used during presaturation pulsing is critical. We have shown that even small changes in power levels can have dramatic effects to nearby resonances, thereby throwing off peak ratios and the performance of automatic assignment software [80,83,84]. Power levels must be carefully calibrated and consistently delivered when using different NMR instruments and probes. Even with careful calibration and near identical hardware, it is not sufficient to use the same parameter settings. For example, two “300 watt” amplifiers may have slightly different linearity or effective attenuation, cable lengths, tune/match parameters, or effecting probe performance (to name but a few possibilities). NMR instrument manufacturers often go to great lengths to achieve uniformity across their systems, but with so many components, different usage patterns (i.e., wear and tear), and vastly different hardware/software settings, there is only so much that can be done. Careful, meticulous, and repeated verification by the user on standard samples can alleviate these concerns and compensates for most, if not all, of the possible spectrometer complications. Calibration of the effective 90° pulse angle by the user, subsequent determination using calibration linearity, and application of a consistent (e.g., 80 Hz gammaB_1_ effective) presaturation pulse eliminates the addition of a “confounder” to the results [82].

While careful instrumental calibration is key to excellent reproducibility for most NMR experiments, it is important to remember that the nondestructive nature of NMR also helps with experimental reproducibility. In particular, NMR samples can be repeatedly measured, stored, and remeasured to validate or confirm earlier findings. It is also possible to perform new types of NMR experiments on the same sample to answer questions that were not previously answered with an earlier experiment. This is because extended storage of biosamples at −80 °C and retesting after freeze/thaw cycles have little to no observable effect on the measured NMR results [85,86]. This fact greatly extends the exploratory potential of NMR, and it allows users to perform an initial quick evaluation of a sample and then to repeatedly return to the same sample for more detailed analysis. Remarkably few groups take advantage of this potential, as most NMR-based metabolomics researchers only conduct fast 1D-^1^H experiments without taking the time to reanalyze the sample using (lengthier) 2D correlation experiments. Indeed, it seems too many NMR users appear to be unaware of the fact that multidimensional NMR offers substantially more resolving power (e.g., 2D ^1^H,^13^C-heteronuclear single quantum correlation spectroscopy (HSQC), heteronuclear multiple bond correlation (HMBC), correlation spectroscopy (COSY), total correlation spectroscopy (TOCSY), etc.) [87,88]. The authors of this article feel that there is an unnecessary push to only use very short, 1D NMR experiments in NMR-based metabolomics. While we certainly understand the desire for fast, cost-efficient experiments in studies involving many samples, one of the great strengths of NMR undoubtedly lies with longer, and/or more complex (i.e., multidimensional) experiments to positively identify known compounds, to characterize novel compounds, or to uncover previously undetected compounds.

**Figure 4:**
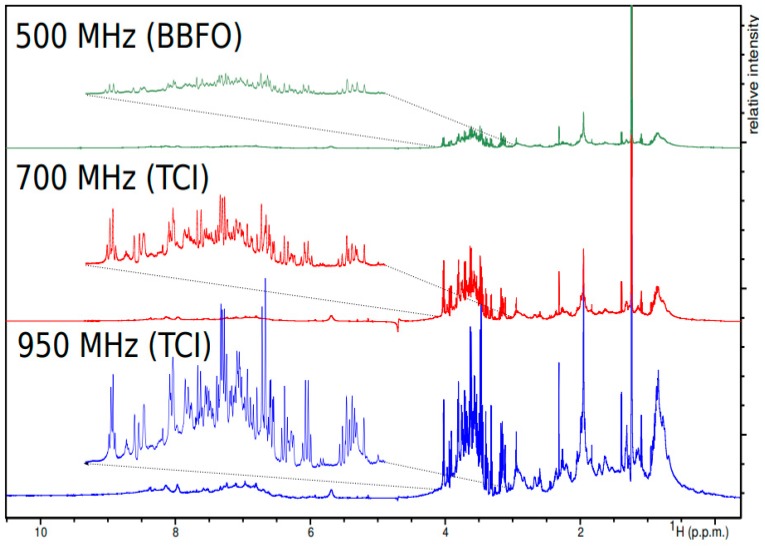
Demonstration of magnetic field strength and probe specificity on spectral resolution of bovine serum recorded with the same parameter set on three spectrometers working at 500, 700, and 950 MHz proton frequencies at 25 °C. The probes used are the Bruker TCI—Triple Resonance CryoProbe on the 700 MHz and 950 MHz instruments and a Bruker BBFO on a 500 MHz magnet.

Despite the many advantages of ^1^H NMR spectroscopy, there are a few limitations that have to be considered when choosing the appropriate NMR techniques. For example, the relatively small chemical shift window or spectral width associated with ^1^H NMR spectroscopy means that there is a greater likelihood of overlapping peaks. Overlapping peaks leads to greater ambiguity in compound identification and quantification. One way of addressing peak overlap is to perform NMR experiments with stronger magnets and higher magnetic fields. This increases spectral dispersion. In 1D NMR, resolution scales linearly with the magnetic field strength of the instrument. Unfortunately, the instrument cost often scales exponentially with the field strength. This cost-scaling reflects the vastly expanded complications and physical limitations of creating and stabilizing the powerful superconducting magnets used in ultra-high-field NMR spectrometers. As a general rule, the “sweet spot” for field strength, resolution, and cost in NMR-based metabolomics is the 600 MHz NMR spectrometer. Figure 4 shows the gain in both sensitivity and resolution that comes with using higher magnetic fields for typical metabolomics samples. 

Aside from using larger magnets to improve spectral resolution, selective excitation techniques combined with multidimensional NMR experiments, such as the selective TOCSY experiment, can also help to improve spectral resolution [89,90,91]. Indeed, most two-dimensional ^1^H NMR spectroscopy experiments can assist in resolving overlapping peaks. This improved resolution means that certain 2D ^1^H NMR experiments can potentially detect and identify more metabolites than any given 1D ^1^H NMR experiment. However, substantial increases in instrument time are required to collect these kinds of 2D spectra. So too is the requirement for experienced NMR spectroscopists who are capable of processing and interpreting the acquired multidimensional data and performing the necessary metabolite identification and quantitation. These constraints have, until recently, limited the uptake of two-dimensional ^1^H NMR in NMR-based metabolomic studies. 

### 2.1. ^13^C NMR Spectroscopy for Metabolomics

Compared to ^1^H NMR spectroscopy, which is characterized by narrow line widths and a relatively narrow chemical shift dispersion (of ∼10 ppm), ^13^C NMR spectroscopy has narrow line widths combined with a rather broad (∼200 ppm) chemical shift dispersion. As a result, ^13^C NMR offers a significant resolution advantage over ^1^H NMR. However, the low natural abundance of ^13^C (∼1.1%) coupled with the inherent low sensitivity of the ^13^C nucleus (compared to other common nuclei observed by NMR such as ^1^H, ^19^F, or even ^31^P) substantially hinders the application of ^13^C NMR spectroscopy to metabolomics. 

Fortunately, there are a number of different NMR approaches that can be used to overcome the low natural abundance of ^13^C as well as its inherently low sensitivity to enhance ^13^C NMR signals. For instance, ^13^C signal intensity can be enhanced by a maximum theoretical factor of four using a ‘distortionless enhancement by polarization transfer’ (DEPT) [92] experiment. DEPT NMR experiments can also be used for molecular identification by spectrally distinguishing between CH_2_ and CH or CH_3_ signals. For instance, the ^13^C NMR spectrum of a DEPT-135 experiment yields CH_2_ peaks with the opposite intensity compared to CH and CH_3_ peaks [93]. It is worth noting that ^1^H signal enhancements from ^1^H/^13^C NOESY nuclei polarization transfer can theoretically reach up to a factor of 1.98, thereby saving a factor of 4 in total scan time. However, this is rarely achieved in practice because there are different contributions of dipole–dipole relaxation mechanisms. 

While ^13^C NMR signal enhancement can be achieved through ingenious spin-manipulation (via the DEPT experiment), it can also be achieved through ^13^C enrichment. For instance, the use of ^13^C-enriched glucose has long been used to label metabolites in microbial metabolomics studies [94] and mammalian cell line studies [94,95], while the use of ^13^C enriched CO_2_ can be used to label metabolites in plant metabolomic studies [96,97]. However, ^13^C enrichment is not usually feasible in mammalian or human studies. To get around this problem, Shanaiah et al. employed a chemical derivatization method or chemical tagging method to enrich unlabeled metabolites with ^13^C [98]. In particular, by adding ^13^C-acetic anhydride to urine or serum samples, it is possible to selectively ^13^C-tag certain classes of metabolites (such as amino acids) through the ^13^C acetylation of amines. This tagging enables rapid collection of ^13^C NMR spectra while at the same time significantly simplifying the NMR spectra of complex biofluids (such as urine) through selective labeling. This chemo-selective tagging method was successfully used to identify and compare amino acids and related metabolites in urine collected from patients with inborn errors of metabolism [99]. We believe this simple and elegant approach for enhancing ^13^C NMR signals could be extended to many other applications in human or mammalian metabolomics.

^13^C signal enhancement is also possible through the use of improved hardware and probe designs. In particular, the use of cryoprobe technology, where the NMR probe and its electronics are cooled to near absolute zero as a way to reduce electronic noise, can lead to a two- to four-fold signal enhancement. For instance, Keun and colleagues employed a ^13^C direct-detect cryoprobe to study drug toxicity in urine via 1D ^13^C NMR [100,101]. This type of cryoprobe permitted the acquisition of useful 1D ^13^C NMR spectra in approximately 15 min, which is similar to the time typically taken to collect a 1D ^1^H NMR spectrum. The resulting ^13^C spectra had much greater spectral dispersion than ^1^H spectra (which is critical in analyzing complex biofluids like urine) while still providing sufficient signal-to-noise to enable analysis. Another approach that can be used to enhance ^13^C NMR signals involves the use of hyperpolarization techniques, which are described in Section 5.2. 

It is important to note that ^13^C NMR is particularly useful for isotope tracking or isotope tracing experiments [98,102,103]. These metabolic experiments allow the direct determination of the source of certain carbons in various biosynthetic pathways as well as further clarification of the precise chemistry involved in various biosynthetic steps. As a result, ^13^C has become the most commonly observed nucleus in experiments involving NMR as well as MS-based fluxomics. Fluxomics is a branch of metabolomics that focuses on determining intracellular metabolic fluxes in living cells [104]. The experiment consists of incorporating isotopically enriched ^13^C molecules in living cells and then quantifying the metabolic activity (i.e., performing the flux measurement) by tracking and quantifying ^13^C isotope propagation through the investigated metabolic network investigated. 1D ^13^C direct detection, or indirect ^13^C detection via 2D NMR techniques such as ^1^H-^13^C HSQC, are often employed in these cell-based NMR experiments.

### 2.2. ^15^N NMR Spectroscopy for Metabolomics

Like ^13^C NMR, ^15^N NMR spectra are characterized by a broad chemical shift dispersion (∼100 ppm) and relatively narrow line widths. However, the direct detection of ^15^N is challenging because of its very poor sensitivity. In particular, its low natural abundance (0.37%) and its low gyromagnetic ratio (7.62 MHz/T) conspire to make the ^15^N nucleus over 262,000 times less sensitive than the ^1^H nucleus. As a remedy, isotopic enrichment combined with ^1^H-mediated enhancement via indirect detection is often the only route to make ^15^N NMR practical. 

**Figure 5:**
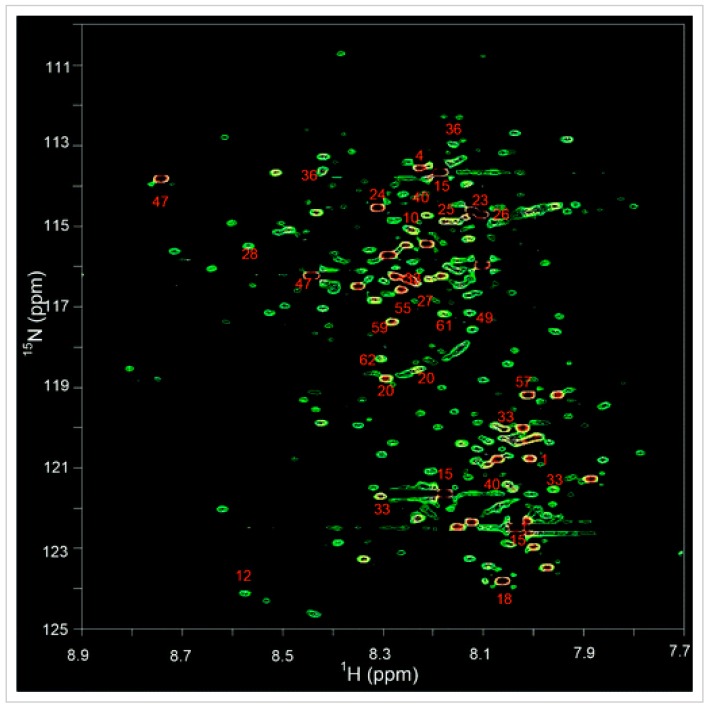
Detection of nearly 200 carboxyl-containing metabolites in urine by 2D ^1^H-^15^N heteronuclear single quantum correlation spectroscopy (HSQC) NMR after tagging with ^15^N isotope containing ethanolamine [46].

Indirectly detected ^15^N NMR spectroscopy is widely used in structural elucidation of proteins [105,106,107], RNA [108,109,110], and DNA [111,112,113,114], but it has not been commonly applied in metabolomics studies. The Raftery group has pioneered the development of ^15^N isotope-based indirect detection for NMR-based metabolomics. Their ingenious concept is based on isotope tagging to expand the pool of quantifiable metabolites [46]. More specifically, Raftery’s approach involves reacting carboxyl-containing metabolites with an ^15^N-ethanolamine tag and then detecting the tagged molecules using ^1^H-^15^N 2D NMR. This approach, like the ^13^C tagging approach described earlier, allows selective tagging of certain metabolite classes that provides further spectral simplification. Typically, this selective tagging approach generates a single peak for each tagged metabolite, effectively suppresses the signal from nontagged metabolites, and significantly adds to the sensitivity and peak dispersion. This can enable the detection of over a hundred quantifiable metabolites from a single class of molecules (i.e., carboxylic acids) using a two dimensional ^1^H-^15^N HSQC experiment (Figure 5). More recently, Raftery’s team has developed a “smart” isotope tag (^15^N-cholamine) to exploit the combined strength of NMR and MS for unknown compound identification and quantitation. ^15^N-cholamine possesses dual characteristics: a ^15^N isotope that enables NMR detection of tagged metabolites with high sensitivity and good chemical shift dispersion as well as a permanent charge that improves MS sensitivity [46,105]. 

### 2.3. ^31^P NMR Spectroscopy for Metabolomics

While ^31^P is nearly 100% abundant, has a relatively wide spectral dispersion and has a sensitivity of 6.6 × 10^−2^ relative to ^1^H. Its utility in metabolomics studies is limited because most metabolites do not contain phosphorus atoms. As a result, ^31^P NMR spectroscopy in metabolomics has mainly been limited to studying a small number of important phosphorous-containing compounds such as phospholipids and nucleoside metabolites (ATP, GTP, NADP, etc.) involved in energy metabolism [47,115].

One approach that potentially expands the utility of ^31^P NMR for metabolomics was recently provided by the Raftery group [116]. Like the ^15^N tagging method described earlier, this approach uses isotope tagging to enable the detection of various hydrophobic compounds. More specifically, this method employs the ^31^P reagent, 2-chloro-4,4,5,5-tetramethyldioxaphospholane (CTMDP), to tag lipid metabolites containing hydroxyl, aldehyde, and carboxyl groups. One-dimensional ^31^P NMR then enables detection of the tagged metabolites with enhanced resolution. This method, which was applied to the detection of a number of metabolites in serum, was shown to be simple, reproducible, and highly quantitative. 

## 3. Two-Dimensional (2D) NMR Spectroscopy

Two-dimensional NMR spectroscopy can be used for many applications including molecular identification, structural elucidation, and kinetic or energetic analysis [117,118,119]. In metabolomics, 2D NMR can be used to overcome the problem of overlapping resonances by spreading the peaks into a second dimension based on another “orthogonal” physical property of the atom or atoms of interest (e.g., a covalently attached neighbor, a relaxation time, a coupling constant, etc.). The additional resolution available through 2D NMR potentially allows one to detect and identify more metabolites than possible with 1D NMR. Indeed, different homonuclear 2D ^1^H-^1^H-NMR experiments, including many variations by total correlation spectroscopy (TOCSY) [120], correlation spectroscopy (COSY) [30,121], and nuclear Overhauser effect (NOESY) experiments [122] along with heteronuclear ^1^H,^13^C single quantum coherence (^1^H-^13^C-HSQC), and heteronuclear multiple bond correlation (HMBC) experiments have been routinely used in metabolomics studies for many years [123,124,125]. Diffusion ordered spectroscopy (DOSY) [126,127] and two-dimensional J-resolved NMR spectroscopy (J-Res) [88] are examples of other 2D NMR experiments that have also been used in several NMR-based metabolomics studies. In the following sections we will describe some of the more popular 2D NMR experiments for metabolomics in more detail. 

### 3.1. Correlation Spectroscopy (COSY)

The single, relayed correlation spectroscopy (COSY) [128] experiment is the simplest of all 2D NMR experiments. It provides information on homonuclear correlations between coupled nuclei (^1^H-^1^H) and has been widely used for molecular identification and for structural elucidation [129,130,131,132]. The COSY pulse sequence consists of a 90° radio frequency (RF) pulse [133] followed by an evolution time (t_1_) then a second 90° pulse, which is followed by a measurement period time (t_2_). Fourier transformation in both the t_1_ and t_2_ dimensions yields a 2D spectrum, in which the cross peaks in the 2D spectrum indicate pairs of nuclei connected by through-bond (3JHH) couplings. The fact that the COSY experiment is relatively simple, fast (often a few minutes), easy to perform, and easy to interpret makes it particularly useful for metabolomics research [134,135,136,137,138]. Moreover, COSY cross peaks, which represent through-bond coupling between coupled nuclei, provide important clues for the identification of unknown metabolites in complex biological mixtures. In other words, COSY experiments (as well as other 2D NMR experiments) enable the identification of both known and unknown metabolites, while 1D NMR experiments are largely limited to the identification of known metabolites. 

There are numerous variations or forms of the COSY NMR experiment [139,140,141]. As a result, one must be careful in choosing the proper COSY variant by carefully considering which type and what physical aspects one wants to observe. For example, the magnitude mode acquisition COSY reduces the complexity of the experiment, but it loses much of the coupling information that other COSY versions allow. It is also worth mentioning that, for very complex mixtures composed of many small molecules (such as urine), the spectral complexity due to spectral overlap is often so great that one quickly loses the inherent advantages of the 2D COSY. 

### 3.2. Total Correlation Spectroscopy (TOCSY) 

TOCSY (total correlation spectroscopy), also known as the homonuclear Hartmann–Hahn (HOHAHA) experiment, is an extension of the COSY experiment, wherein the chemical shift of a given nucleus is correlated with the chemical shift of other nuclei within the total (or near total) spin system of a given compound. The TOCSY spectrum shows the cross peaks not only for short-range (e.g., 3JHH) coupled protons but also for protons that are connected by a chain of scalar couplings (e.g., four or more covalent bonds away). For instance, if both proton A and C are coupled with proton B (but A and C do not directly couple with each other), then the TOCSY spectrum would show a correlation of A with both B and C, while the COSY spectrum would just show the coupling between A and B and that between B and C.

While the 2D TOCSY experiment takes a relatively long time to collect, the 1D TOCSY is very quick (often a minute or two) and produces a relatively simple 1D NMR spectrum that is more easily analyzed. The 1D TOCSY is characterized by a spectrum in which only signals appear from those nuclei that are in the same spin system as the excited signal or signals. The 1D TOCSY experiment is particularly useful when there is considerable overlap in the NMR spectrum or when one wishes to quantify highly overlapped metabolite species (100). The 1D TOCSY was first shown to be useful in a metabolomics setting with the analysis of low-concentration metabolites in honey samples where carbohydrate signals were extremely strong and dominated the NMR spectra [91]. Selective excitation TOCSY experiments are another variant of the TOCSY experiment that can be used to resolve the spectral overlapping problem and to aid in metabolite identification [90,142,143] 

### 3.3. 2D J-Resolved Spectroscopy (J-Res)

The 2D J-resolved spectroscopy (J-Res) experiment is one of the oldest of all 2D NMR experiments. It was initially introduced by Ernst et al. as a means to display both J-couplings and chemical shifts in the same 2D NMR spectrum [144]. As with other 2D experiments, the J-Res experiment simplifies spectral assignments by increasing the peak dispersion compared with a conventional 1D NMR experiment [145]. 

Because of its speed relative to other 2D methods, 2D J-Res NMR spectroscopy has become a popular method for a wide range of NMR-based metabolomics studies [88,146]. For example, J-Res NMR spectroscopy has been employed to resolve overlapped resonances of metabolites and for metabolite identification in human biofluids such as urine, blood plasma, and cerebral spinal fluid [147,148,149]. It was reported that plasma J-Res NMR spectra were simpler and yet contained much more information than the corresponding 1D Hahn spin-echo spectra. J-Res NMR spectroscopy has also been shown to have similar advantages when employed with different types of biological samples such as hemolymph from tobacco hornworm larvae (*Manduca sexta*) [150]. The basic methodology of 2D J-Res NMR spectroscopy, along with optimized spectral acquisition parameters and specific recommendations for optimal data processing in the context of metabolomics applications, has recently been reviewed [146].

The extended experimental time is one of the main disadvantages of 2D NMR experiments, especially with metabolomic studies that involve a large number of samples. Several approaches have been developed to shorten the acquisition time of multidimensional NMR experiments, including the J-Res experiment [30,151,152]. For instance, Frydman et al. optimized and developed a single-scan 2D NMR method, called planar imaging [153,154], which has been integrated with the 2D J-Res experiment. This has reduced the J-Res acquisition time to less than one minute [155]. However, these experiments suffer from extremely low sensitivity and require very high metabolite concentrations that are not practical for most metabolomics studies. An alternative to the 2D J-Res experiment is the 1D J-Res. In particular, the proton-decoupled projected 1D J-Res experiment (p-JRES) offers some of the advantages of the 2D J-Res experiment along with the speed advantages of a typical 1D experiment [156].

### 3.4. Heteronuclear Single Quantum Correlation Spectroscopy (HSQC)

Bond correlation spectroscopy (COSY and TOCSY-like spectroscopy) is not limited to homonuclear correlations; therefore, it can also be used for measuring heteronuclear correlations. Furthermore, heteronuclear correlation experiments can be used to enhance the signal coming from a lower sensitivity nucleus by transferring the nuclear spin polarization from the more sensitive nucleus via J-coupling (i.e., between two different types of NMR active nuclei). Such nuclear spin polarization transfer from nuclei with large Boltzmann population differences (mainly ^1^H) to nuclei with a low Boltzmann population difference (e.g., ^13^C, ^15^N) is called insensitive nuclei enhanced by polarization transfer (INEPT). Here, the magnetization from the more sensitive nucleus (usually ^1^H) is transferred to the less sensitive nucleus, such as ^13^C or ^15^N, and then transferred back to ^1^H for direct observation. The general feature of the INEPT-based HSQC experiment is the mapping of the chemical shift of one nucleus (such as ^1^H) detected in the directly measured dimension and the chemical shift of the other nucleus, such as ^13^C, recorded in the indirectly measured dimension. 

An ^1^H,^13^C-HSQC spectrum maps the chemical shifts of proton and carbon atoms that are directly bonded, providing only one cross-peak for each H–C coupled pair. Likewise, an ^1^H,^15^N-HSQC spectrum maps the chemical shifts of directly bonded proton and nitrogen atoms yielding one cross-peak for each H–N coupled pair (see Figure 5). HSQC is a very useful experiment for resolving and assigning overlapping proton signals, particularly for metabolite signals arising from complex biofluid mixtures. HSQC experiments are also among the most important and common experimental techniques in biomolecular NMR for the assignment of protein backbone and side-chain NH signals [157,158,159,160]. Heteronuclear, multidimensional NMR experiments utilizing the sensitivity of the ^1^H nucleus are very effective at reducing the experimental time needed to measure nuclei with low natural abundances and/or low NMR sensitivity such as ^15^N and ^13^C. 

**Figure 6:**
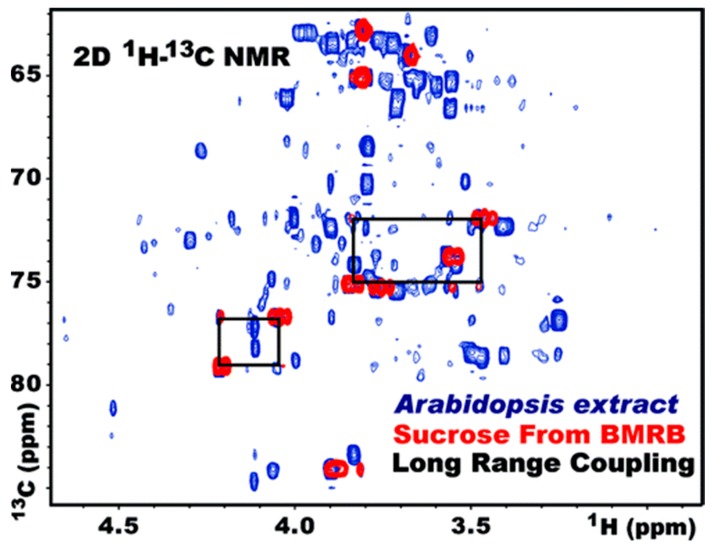
2D ^1^H−^13^C HSQC NMR spectrum of sucrose from the Biological Magnetic Resonance Data Bank (red) overlaid onto an aqueous whole-plant extract from *Arabidopsis thaliana* (blue) [161].

As noted earlier, the long experimental acquisition time is the main drawback of 2D NMR, especially for NMR-based metabolomics. Furthermore, because of the large number of metabolites with a broad concentration range, 2D NMR spectra need to be collected with high-resolution settings. The greater the resolution required, the longer the experiments take. Fortunately, a number of novel pulse sequences have been developed to help address these problems for HSQC spectra. One example is a 2D ^1^H,^13^C-HSQC strategy for fast metabolite quantification (FMQ). FMQ has been employed to identify and quantify 40 of the most abundant metabolites in biological samples, with accurate metabolite identification and concentration determination with spectra collected in as little as 12 min [161] (Figure 6).

Heteronuclear multiple-quantum correlation spectroscopy (HMQC) is another two-dimensional correlation experiment similar to the HSQC experiment. HMQC provides virtually the same type of correlation as in HSQC, but it uses a different approach to transfer magnetization from ^1^H to the heteronucleus. HMQC has been less attractive for metabolomics applications as it typically gives much broader peaks than HSQCs. However, development of a rapid acquisition (SOFAST) approach has increased the interest to use SOFAST-HMQC for fast data collection [162].

### 3.5. Heteronuclear Multiple Bond Correlation (HMBC) Spectroscopy

The HMBC is another common 2D heteronuclear experiment that correlates the chemical shifts of two different types of nuclei (i.e., ^13^C and ^1^H) similar to HSQC or HMQC experiments. However, unlike HSQC or HMQC, the HMBC experiment reveals correlations between nuclei that are separated by two or more chemical bonds. To eliminate the single bond correlation, a low-pass filter is used, where only smaller J-couplings are optimized for detection. HMBC, thus, eliminates single C–H bond correlations and is useful for the assignment of signals from quaternary and carbonyl carbons that are not detected in HSQC and HMQC experiments. For molecular identification through complete signal assignments and for chemical structure elucidation, a combination of HMBC with HSQC or HMQC experiments is commonly used. The utility of ^1^H-^13^C HMBC in metabolomics can be seen in a paper published by Bernin et al. [163]. Using a 900 MHz NMR, these authors acquired an HMBC spectrum of urine and were able to easily distinguish some of the aromatic peaks of hippurate, phenylacetylglycine, and histidine.

## 4. NMR Databases and Software for Metabolite Identification

The identification of metabolites by NMR requires an appropriate set of reference chemical shifts or reference spectra for pure compounds. By comparing the observed spectra or observed chemical shifts, peak intensities, and/or coupling patterns to these reference spectra, NMR spectroscopists can identify and even quantify compounds from the measured NMR spectra. Ideally, these reference spectra need to be available for all measured nuclei (^1^H, ^13^C, ^15^N, and ^31^P), for all measured types of NMR spectra (1D-^1^H, 1D-^13^C, 2D COSY, 2D TOCSY, 2D J-Res, 2D HSQC, 2D HMQC, and 2D HMBC), for all detected metabolites (about 1000 compounds), and for all of the commonly measured spectrometer frequencies (400, 500, 600, 700, 800, and 900 MHz and even 1 GHz). If appropriate reference spectra and sufficiently sophisticated software tools are available, a trained individual can identify and quantify all the metabolites in a relatively simple biofluid (such as serum) in about 30 min [81]. 

Historically, NMR databases of most chemical compounds were compiled and kept in books. However, the size and complexity of today’s NMR spectral databases—especially those needed for metabolomics—has required that they be placed on the web. Thanks to the efforts of a number of metabolomics labs from around the world, there are now several high-quality, web-based NMR spectral databases containing reference NMR spectra for hundreds of metabolites collected over a wide range of spectrometer frequencies and for a diverse range of nuclei. Many of these databases are freely accessible by the public, including the Human Metabolome Database (HMDB) [164,165], the Biological Magnetic Resonance Data Bank (BMRB) [166], the Madison-Qingdao Metabolomics Consortium Database (MMCD) [69], the NMRShiftDB2 database [167], and the AIST spectral database in Japan (https://sdbs.db.aist.go.jp). 

Here we will briefly describe a few of these databases in more detail. The Human Metabolome Database (HMDB, www.hmdb.ca) [165,168] is becoming the de facto standard reference database for most studies involving mammalian or human metabolomics. It is a freely available electronic database, created and housed at the University of Alberta, containing information on >114,000 metabolite entries gathered from thousands of books, journal articles, and electronic databases. Each metabolite entry contains more than 100 data fields including descriptions, chemical formulae, names, synonyms, structures, physical and chemical properties, pathways, reactions, enzymes, transporters, disease links, and literature references. In addition to its comprehensive literature-derived data, the HMDB also contains an extensive collection of experimental metabolite concentration data compiled from hundreds of MS and NMR metabolomic analyses performed on urine, blood, and cerebrospinal fluid samples. This is further supplemented with thousands of NMR and MS spectra collected on purified reference metabolites. Currently, the HMDB has 3897 NMR spectra for 2391 compounds. This includes 2862 1D ^1^H and ^13^C spectra and 1035 2D ^1^H-^13^C-HSQC spectra collected at 500 and 600 MHz. 

The Madison-Qingdao Metabolomics Consortium Database contains information on more than 20,000 compounds, including NMR data (173). It is maintained by the National Magnetic Resonance Facility at the University of Wisconsin (Madison) with the aim of supporting high-throughput NMR and MS approaches for the identification and quantification of metabolites present in biological samples [69]. Each metabolite entry in the MMCD is supported by information from an average of 50 separate data fields, which provide the chemical formula, names, synonyms, structure, and physical and chemical properties. The MMCD contains standardized experimental NMR data (1D-^1^H, 1D-^13^C, DEPT, TOCSY, and ^1^H-^13^C-HSQC) for 794 compounds collected at 400 MHz along with literature-derived NMR spectra data for another 1156 compounds. The same collection of experimental NMR spectra for the same set of 794 compounds is also housed in the BMRB metabolite database (172). 

The NMRshiftdb2 database (http://nmrshiftdb.nmr.uni-koeln.de/) is primarily a spectral database of organic compounds prepared for (and by) organic chemists [169]. Originally developed by Stefan Kuhn and Christoph Steinbeck, this database currently contains 1D-^1^H and ^13^C NMR spectra for more than 40,000 different molecules. However, most of these NMR spectra were not collected in water (which is essential for metabolomic studies), and most of the compounds are not biological (i.e., not metabolites). However, with the growing interest in metabolomics, more and more metabolite spectra are being deposited into this database. 

The National Institute of Advanced Industrial Science and Technology (AIST) in Japan maintains the Spectral Database for Organic Compounds (SDBS, http://sdbs.db.aist.go.jp/sdbs/cgi-bin/cre_index.cgi), an integrated spectral database for organic compounds, which includes six different types of spectra: EI-MS, FT-IR, 1D ^1^H-NMR, 1D ^13^C-NMR, Raman, and ESR. Currently the SDBS contains data on 34,600 compounds with 15,900 ^1^H-NMR spectra and 14,200 ^13^C-NMR spectra. As with NMRshiftdb2, most of the compounds in the AIST database are not metabolites.

NMR spectra databases are only useful if they can be used to help with compound identification and/or quantification. In many cases, NMR spectroscopists simply look up their measured chemical shifts online to identify their compounds. Others will use commercial software (that usually has its own proprietary NMR spectral databases), such as the well-known Chenomx NMRSuite, to identify compounds. Over the past five to six years, several freely available software tools have been developed to facilitate NMR-based compound identification for metabolomics. Most of these have been designed for deconvoluting 1D ^1^H NMR spectra. Some of the first open-access programs to appear include Batman [65] and Bayesil [66]. Batman uses Bayesian methods and a small, local NMR reference database to deconvolute 1D ^1^H NMR spectra. It is capable of identifying 15–20 metabolites in defined metabolite mixtures, although it is relatively slow (up to several hours per spectrum). Bayesil, which is available as a web server, makes use of probabilistic graphical models (PGMs), similar to hidden Markov models (HMMs), to perform 1D ^1^H NMR spectral deconvolution. It is capable of automatically identifying and quantifying 45–55 compounds in biofluids such as serum, plasma, and CSF in about 5–6 min. However, Bayesil is limited in the type of biofluids and spectrometer frequencies (only 500 and 600 MHz) with which it can work. Several other 1D NMR spectral deconvolution programs have also recently appeared, including the automated quantification algorithm (AQuA) [67] and automatic methods for identification and quantification of metabolites called ASICS [70] and rDolphin [68]. These methods appear to perform quite well and, in some cases, are even faster than Bayesil. 

Another important advance in NMR spectral deconvolution has been the development of automated lipoprotein and small molecule characterization in whole blood samples [170]. This concept has been commercialized by several companies including Vantera Inc., Liposcience Inc., Labcorp, and Nightingale, a Finnish biotech company. Nightingale now offers a fully automated service and performs large-scale (hundreds of thousands of samples) NMR profiling for the UK BioBank. Automated spectral assignment and metabolite quantification algorithms represent one of the most important developments for NMR-based metabolomics. This is because it moves NMR into the realm of near complete automation, something that has not yet been achieved with mass spectrometry based metabolomics. 

A novel approach for the automated and accurate identification of urine metabolites was recently introduced [171]. This has been achieved by constructing a large number (ca. 4000) of artificial urine samples where the concentrations of the most abundant metabolites and of several inorganic ions were varied. This algorithm can accurately predict the chemical shifts of 90 different spin systems. This predictor not only allows for facile identification and quantitation of many metabolites whose signals are clearly visible in the NMR spectrum, it also provides estimates of the concentrations of 11 NMR “invisible” inorganic ions (software available for testing at http://150.217.146.252:8080).

In addition to these 1D NMR tools for spectral assignment and deconvolution, several programs have been developed to deconvolute 2D NMR spectra. In particular, the Bruschweiler group has developed an algorithm, called complex mixture analysis by NMR (COLMAR), that enables improved identification of metabolites via 2D TOCSY or 2D HSQC spectra curated from the BMRB and HMDB [172]. Another program, called MetaboMiner, enables automated or semi-automated metabolite identification from 2D NMR spectra (TOCSY or HSQC) using a reference spectral database compiled from the HMDB [173]. However, 2D NMR spectra are inherently more difficult to derive quantitative information from than 1D NMR spectra. As a result, the currently available set of 2D spectral deconvolution software programs are mostly limited to metabolite identification only. 

## 5. New NMR Methods in Metabolomics

NMR is a constantly evolving field, and there are a number of new and exciting techniques that are finding their way into NMR-based metabolomics. In this section we will highlight a few of these including high-resolution magic-angle sample spinning (HRMAS), hyperpolarization methods. ultrafast 2D NMR methods, pure-shift NMR techniques, and hybrid NMR approaches. 

### 5.1. High-Resolution Magic-Angle Spinning NMR Spectroscopy (HRMAS)

Intact tissue samples are far too inhomogeneous to be analyzed with standard solution NMR methods. Typically, the resulting spectra consist only of broad and unresolved resonances because there are strong dipolar coupling effects, chemical shift anisotropy, and inherent magnetic susceptibility differences. Although, the main reason for the broad lines in tissue samples is the presence of magnetic susceptibility gradients [174,175,176]. Thus, when done properly, NMR can also be performed on semisolid tissue samples [177,178,179,180,181,182] with the use of high-resolution magic-angle spinning HRMAS spectroscopy [31,183,184,185]. Line broadening due to the dipolar interaction and chemical shift anisotropy can be averaged by spinning samples at high speed at the ‘magic’ angle (an angle of 54.74°) with respect to the permanent magnetic field. For tissue or gel-like samples, HRMAS spectra can be obtained with a resolution comparable to that of solution-state NMR spectra. Using HRMAS, tissue samples can be examined without the need for sample extraction or other sample preparation steps [186,187]. 

HRMAS is very helpful for metabolomics studies. It provides a close link between the metabolic profile obtained for biofluids and the histology measured using tissue. Importantly, with HRMAS, the microstructure of the tissue specimens remains intact even after NMR analysis. Decomposition of the tissues does not occur during NMR measurements as the samples are typically analyzed at low temperatures in less than ~20 min [188]. Because of HRMAS’s nondestructive, nondisruptive nature, the same tissue specimens can be subjected to histopathology and other analyses for further validation. As a result, HRMAS is increasingly being used in human metabolomic studies that involve investigation of cancer tissues such as kidney [188], liver [189], brain [190], and testicular tissues [191].

Some of the most exciting developments in HRMAS have occurred because of recent probe design advances. In particular, microprobes for magic-angle spinning (μMAS) of sub-microgram specimens with high-resolution (<0.01 ppm) capability have recently appeared [192]. These permit the site-specific metabolomic characterization of different anatomical regions of plants and tissues [193]. While sample preparation can be difficult, the capabilities of μMAS probes gives NMR spectroscopists the opportunity to systematically explore tissue-specific metabolism with nearly equal precision as imaging mass spectrometry [194]. An interesting example of how HRMAS spectroscopy can be employed in modern metabolomic experiments was recently described [195]. In this study, HRMAS was employed in conjunction with principal component analysis (PCA) and orthogonal projection to latent structure with discriminate analysis (OPLS-DA) to examine the metabolic profiles of 127 tissue samples collected from patients diagnosed with colorectal cancer. These samples were then compared with 47 samples collected from healthy control subjects. Sixteen tissue metabolites were found to be closely correlated with the staging or degree of advancement of the colorectal cancer. In another similar study, HRMAS was used to characterize meningioma biopsies as a potential diagnostic tool to distinguish between typical meningiomas and benign tissues [196]. In another paper, HRMAS was used to monitor the impact of ischemia on the metabolic profiles of 162 human liver samples collected during and up to 6 h post-surgery [197]. The authors reported a significant change in the tissue metabolome as a function of intraoperative warm ischemia and post-resection cold ischemia time. Clear variations in the concentrations of 16 metabolites could be observed, showing that HRMAS spectroscopy could be quite useful in assessing tissues and tissue damages prior to organ transplantation.

### 5.2. Hyperpolarization Methods

#### 5.2.1. Dynamic Nuclear Polarization (DNP)

Dynamic nuclear polarization (DNP) is a technique that was originally developed in the 1950s to enhance the NMR signal intensities in low-field magnets; however, recent advances have allowed DNP to be routinely conducted with high-field NMR instruments [198,199]. The fundamental principle behind DNP involves transferring the Boltzmann polarization by saturating the electron resonances to transfer the polarization of the electron spins to the nuclear spins. For example, in a variant of DNP known as dissolution DNP (D-DNP), a solid, frozen sample that has been cooled to about 1.5 K is polarized in the presence of microwave-irradiated free-radicals. This induces a temporary hyperpolarization in the spin-active nuclei (usually naturally abundant ^13^C or ^15^N, but also ^1^H) through a transfer of the spin polarization from electrons to the nuclei of interest. After the polarization transfer step is completed, the sample must be rapidly melted (hence the term dissolution) and transferred to the NMR spectrometer to collect the enhanced (>1000-fold) NMR signals in the liquid state [200]. D-DNP eliminates the need to use expensive isotope labeling and also allows for the detection of metabolites present in lower concentrations.

In principle, the sensitivity enhancement is on the order of the electron and nuclear gyromagnetic ratios (~2600 for electron-^13^C spins and ~657 for electron-^1^H spins), which provides a theoretical enhancement of three orders of magnitude. Signal enhancement is typically measured by comparing the NMR signal intensities before and after DNP. Four different mechanisms, namely the solid effect (SE) [201,202], the Overhauser effect (OE) [203], the cross effect (CE) [204,205], and thermal mixing (TM) [206,207], are currently used in liquid and solid DNP experiments. While the cross effect is the most relevant to solid DNP, the Overhauser effect (OE) is commonly used for liquid-state DNP-NMR experiments. Although the maximum theoretical enhancement values are difficult to achieve [208], substantial enhancements of ^15^N and ^13^C spectra of three orders of magnitude are commonly achieved [209,210]. 

#### 5.2.2. Applications of DNP in Metabolomics

Because of the differences in the natural abundance of NMR-active nuclei, some nuclei are more useful for metabolomics studies than others. For instance, at natural ^13^C abundance (1.109%), metabolomics studies based on heteronuclear ^13^C-^1^H NMR techniques are severely limited by sensitivity. The use of dissolution DNP (D-DNP) for detecting ^13^C nuclei has helped overcome this problem, as recently presented in a study involving the metabolomic analysis of tomato extracts [210]. Dissolution DNP, invented in 2003 by Ardenkjaer-Larsen [200], uses a specially developed mixture of free radicals and solvents (called DNP “juice”) and has been shown to give resonance signal enhancements of up to 10,000 times. As a result, D-DNP has been used in a number of metabolomic applications including in vivo studies of cancer metabolism [211,212], in-cell NMR spectroscopy [213], and many other applications including medical analyses [214].

#### 5.2.3. Parahydrogen-Induced Polarization (PHIP) and Signal Amplification by Reversible Exchange (SABRE)

Recently, a new type of hyperpolarization approach has been developed based on parahydrogen techniques such as signal amplification by reversible exchange (SABRE) and parahydrogen-induced polarization (PHIP). The PHIP technique has found several applications in the fields of chemistry and medicine [215]. In chemistry, PHIP has been successfully used to study short-lived reaction intermediates, to investigate catalytic mechanisms, and to determine chemical process kinetics [216]. In the medical field, PHIP has been used to facilitate studies of tumors and to facilitate the examination of the metabolism of physiologically relevant substances [215]. The PHIP technique generates a continuous flow of hyperpolarized gas, which enables polarization transfer to the nuclei (and compounds) of interest. One of the most convenient gases for this purpose is nontoxic propane (a crucial feature for biomedical applications), and it can be easily produced in a hyperpolarized state by the hydrogenation of propene with parahydrogen [215]. One limitation of PHIP-hyperpolarized propane gas is its short hyperpolarization lifetime [217] (Kovtunov et al., 2014) [217]. Another key limitation of conventional PHIP is its [217] requirement for the pairwise addition of parahydrogen to an unsaturated substrate [215].

Ten years ago, in 2009, Duckett and co-workers [218,219] reported a nonhydrogenative technique, called signal amplification by reversible exchange (SABRE), where the parahydrogen as well as a to-be-hyperpolarized substrate molecule underwent simultaneous chemical exchange on a metal (typically iridium) complex. In the last few years, the SABRE technique has found applications as a tool to investigate catalytic mechanisms of reactions, molecular imaging, ^1^H MRI contrast analysis [220,221] using parahydrogenated glucose derivatives as ^13^C-hyperpolarized probes for MRI [222], continuous polarization [223,224,225,226] via membranes [227], as well as polarization storage via long-lived singlet states [215,223,225,228]. Importantly, the SABRE technique allows one to hyperpolarize different ^13^C and ^15^N (at natural abundance) molecules simultaneously. This opens up new possibilities to measure metabolites at low concentrations (drugs, metabolites, etc.), as shown by Reinieri et al. [222].

### 5.3. Fast NMR Methods

As repeatedly mentioned throughout this review, long acquisition times are a major drawback for 2D NMR experiments in metabolomics. However, several methods have been developed that now allow NMR spectroscopists to obtain fast and accurate 2D NMR spectra in a timeframe comparable to that needed to collect 1D NMR spectra. One such approach is nonlinear sampling (NLS), which reduces the number of indirectly detected increments while maintaining a good resolution [155,156]. However, the number of indirect increments can only be reduced by about twofold for most 2D NMR experiments, otherwise spurious signals or artifacts will be introduced in the 2D NMR spectra [157]. Other fast 2D methods are Hadamard spectroscopy [229,230], which requires prior knowledge of the specific metabolites in the biological samples and their chemical shifts. Here, the region of spectral interest is selectively excited and then followed by a Hadamard transformation. While there is tremendous appeal and excitement about these “fast 2D” methods, all of them suffer from some drawbacks. In particular, all of them are quite complex to implement in high-throughput metabolomics studies, and most of them are not particularly quantitative (due to the very special acquisition or transformation conditions).

One promising technique that is even faster than fast 2D NMR is ultrafast 2D NMR spectroscopy. Ultrafast 2D NMR is based on the idea of obtaining the indirect increments in a single experiment by physically isolating vertical slices of the sample (using pulsed field gradients) and then treating each slice as a separate component of the total data acquisition. The raw 2D data matrix is then processed using specialized software tools to regenerate the full 2D NMR spectrum. This elegant and truly innovative approach to doing 2D NMR reduces the acquisition time by several orders of magnitude [231]. Lower sensitivity is the major drawback in this approach. In order to overcome the sensitivity issue, multiscan hybrid ultrafast 2D NMR methods have been developed, which provide good sensitivity in a competitive amount of time. With the possible addition of hyperpolarized NMR for signal enhancement, ultrafast NMR appears to hold considerable promise for NMR-based metabolomics studies [231,232].

### 5.4. Pure-Shift NMR

Indirect spin–spin (J) couplings and chemical shifts are the two major characteristics that define the NMR spectra of nearly all molecules. Both characteristics are vital to the elucidation of structures of unknown metabolites. However, peak multiplicity due to J couplings adds to the spectral complexity of biological mixtures. While the suppression of heteronuclear J couplings has been relatively straightforward in the field of NMR spectroscopy, the removal of homonuclear J couplings has been somewhat more challenging. J-resolved 2D NMR spectroscopy was the first experiment to achieve homonuclear broadband decoupling [145]. Although it has a number of limitations, 2D J-resolved experiments still continue to be widely used in metabolomics studies [59,88,156]. A newer approach, called pure-shift NMR spectroscopy [233,234,235,236,237,238], has emerged and achieves even better decoupling and even greater spectral simplification. Indeed, pure-shift NMR promises significant benefits for the analysis of complex mixtures through improvements in both resolution and sensitivity [239,240]. 

In pure-shift NMR, decoupling is achieved by combining novel NMR pulse sequences with innovative data acquisition and data processing techniques. In these experiments, a portion of the free induction decay signal is collected for each increment of a t1 evolution period. The increments are then stitched together to form an interferogram, which can then be transformed to give a broadband homo-decoupled spectrum [241]. Based on this principle, a number of different pure-shift NMR techniques have been developed that have focused on reducing the overall experimental time [233,234,242,243]. However, the need to perform two-dimensional experiments to get small chunks of useful data tends to limit the general utility of these experiments in metabolomic applications. Real-time experiments that involve alternate J refocusing and data collection allow one to obtain multiple chunks of data in a single experiment. This approach could greatly speed up the time needed to perform a pure-shift NMR experiment [244,245,246]. 

While pure-shift NMR offers a number of “theoretical” benefits to NMR-based metabolomics (such as improved spectral resolution by collapsing J-coupled multiplets to singlets), a major bottleneck to pure-shift NMR has been the general loss of sensitivity. Traditionally, the incorporation of selective J refocusing pulse sequence elements for homonuclear decoupling is accompanied by a significant loss of sensitivity. Recently, advances have been made to overcome this challenge. For example, the real-time BIRD (bilinear rotation decoupled) HSQC, proposed by Paudel et al., achieves improved spectral resolution without sacrificing sensitivity [244]. Most recently, a real-time pure shift HSQC-SI sequence optimized for metabolomics studies has been shown to have a ~40% to 50% sensitivity enhancement over conventional pure-shift NMR experiments [240]. As a result, efforts to apply pure-shift NMR methods to metabolomics studies are now underway [240,247,248].

### 5.5. LC-NMR and Other Hybrid NMR Approaches

Liquid chromatography NMR (i.e., LC-NMR) is not necessarily a new hybrid NMR technique, but the trend towards more and more hybrid NMR techniques in metabolomics is new and important as it greatly enhances the capabilities of NMR-based metabolomics. Combining liquid chromatography with NMR spectroscopy (i.e., LC-NMR) allows one to take advantage of the strengths of modern (HPLC, UPLC) chromatographic separation techniques to greatly simplify the complexity of biological mixtures. In many cases, the spectral complexity of biofluids such as urine or fecal water extracts is so great that it is not possible to identify or quantify many of the compounds detected via 1D or 2D NMR spectroscopy [249]. By using chromatography to select different fractions from an initially complex mixture and to collect NMR spectra on these now-simplified fractions, it is often possible to achieve much more complete spectral assignments for the original mixture [250,251]. LC-NMR can be done either off-line (using fraction collectors and NMR tubes) or in-line (using flow probes) with in-line techniques being faster and simpler. LC-NMR is not without some limitations. For instance, large solvent signals from the LC mobile phase can obscure lower intensity signals, while sample dilution caused by the chromatography step reduces overall sensitivity. Other hybrid NMR techniques also exist such as LC-SPE-NMR, where LC-NMR is combined with solid phase extraction (SPE). This approach can help address some of the aforementioned limitations of LC-NMR and can significantly improve metabolite or natural product identification [252]. LC-NMR can also be combined with mass spectrometry (LC-NMR-MS) to perform structure elucidation of novel compounds [253].

## 6. New Developments with NMR Equipment 

As highlighted in the previous section, NMR methods developed by chemists or physicists are constantly emerging to overcome some of inherent instrumental limitations of NMR. NMR engineers are also working to develop new NMR technologies that improve sensitivity, enhance resolution, or accelerate spectral acquisition. Some of the new NMR technologies that are emerging or just on the horizon include very high field magnets and substantially better NMR probes.

### 6.1. NMR Magnets 

Sensitivity and resolution in NMR are intimately tied to magnetic field strength. In particular, signal-to-noise (S/N) or sensitivity increases with the field strength by a factor of ~B_o_^3/2^. Additionally, resolution improves linearly with the field strength (for 1D NMR) and by the square of the field for 2D NMR. No matter which way one looks at it, higher fields lead to better results. As a result, considerable effort is going into the development of ultra-high-field NMR spectrometers. In particular, ultra-high-field spectrometers are currently being developed using new superconducting materials that not only increase the strength of NMR magnets but also reduce the need for costly helium pumped/refrigerated 2.2 K inner chambers. Presently, 1 GHz and 950 MHz are commercially available, and 1.2 GHz magnets are expected to be delivered in 2019 or 2020. These very high field magnets offer better resolution with higher sensitivity allowing the detection of more metabolites in a single experiment than lower field magnets (see Figure 4). Recent advances in magnet technology have also led to the development of maintenance-free cryostats (e.g., Bruker Aeon systems), where there is no liquid N_2_ shield, and the liquid helium is recycled automatically. One drawback for such a maintenance-free system is the annual or semiannual costs of cold-head maintenance to support the helium recycling. However, for laboratories with unstable helium supplies and/or high delivery costs, these kinds of self-recycling systems can be a significant advantage. 

The space required for NMR spectrometers (i.e., the magnet footprints) has also been substantially reduced with the introduction of actively and/or passively shielded magnets. Currently, all of the highest field instruments including 900 MHz, 950 MHz, and 1 GHz magnets are shielded. As a result, their footprints are greatly reduced. This situation is quite different from just 10–15 years ago when 800 MHz spectrometers often required their own building. While the advantages and appeal of ultra-high-field NMR instruments is quite significant, one does have to remember that instruments with ultra-high-field magnets are prohibitively expensive to acquire and expensive to maintain. As a result, most reported NMR-based metabolomics studies use 500 and 600 MHz NMR spectrometers. Instruments at these field strengths are affordable, commonly available, and offer a good compromise between resolution, sensitivity, and cost. The 500–600 MHz instruments are shielded and make their footprint small and robotic sample handling systems much more feasible (i.e., the stray field from higher field systems can make some sample handling systems unusable).

### 6.2. NMR Probes 

The NMR probe contains all the electronics, transmitting and receiving coils, and other hardware such as shimming, locking, and tune/match systems needed to send and receive RF radiation into an NMR sample. Probe design and development has steadily improved, enabling new experiments to be implemented that can detect a wider range of nuclei with ever greater sensitivity. While probe developments were quite spectacular during the early 2000s, much of this has leveled off in terms of the rate and impact of the improvements. Indeed, most probe advancements over the past three to five years have been more incremental rather than truly “game-changing.”

A number of probe advancements over the past decade have greatly improved the situation for NMR-based metabolomics. For example cryogenically cooled probes or cryoprobes [100] can increase signal sensitivity by a factor of three to four, while some of the latest microprobes or microcoil probes not only enhance sensitivity but also reduce the required sample size down to few microliters. Indeed, microcoil NMR probes have been used successfully in many metabolomics studies of urine and serum with significant improvements in mass sensitivity compared to standard 5 mm probes [254]. A few examples of common vertical geometry microprobes are the 1 mm TXI and 1.7 mm TXI probes from Bruker. Of course, the microcoil probes require special low-volume NMR tubes that need only 20–50 uL of sample. A challenge in working with such small-volume tubes is the placement and removal of liquid samples, removal of bubbles, and cleaning of the tubes. Given their fragility and the time necessary to clean these tubes, most labs now treat these small NMR tubes as single-use, disposable glassware products.

Recently, a double-tuned probe circuit has been developed to utilize heteronuclear hyperpolarization at low fields. This development now allows one to carry out double-resonance NMR with single-channel consoles [255]. In addition to standard static probes, which require NMR tubes to be dropped in and retrieved one at a time, the development of flow probes has opened the door to automated, direct-injection, NMR-based analyses [256]. Other probes developed over the past one or two decades that are commonly used in metabolomics include double-resonance broad band probes (often called BBO and BBFO probes), which are widely used in HMBC or HSQC experiments (see above), as well as triple resonance probes such as the Bruker triple-resonance broad band probe (TBO) and triple resonance probe (TXI) are also available. These probes are commonly found in many NMR labs (particularly protein NMR labs) and provide the opportunity to pulse up to three or four nuclei in a single experiment. 

## 7. Limitations of NMR in Metabolomics 

Low sensitivity has always been the primary limitation of NMR spectroscopy. While NMR has no theoretical limit in sensitivity, as one can always increase the number of acquisition scans, the realistic (time-dependent) detection limits are still in the low-micromolar to high-nanomolar range. 

Although significant signal enhancement using higher magnetic fields, cryo-probes, and digital signal processing has improved the situation, many important, low-abundance metabolites still cannot be detected with today’s NMR technology. For example, it is widely known and acknowledged that there are several thousand measurable or detectable metabolites in human biofluids, yet only a few hundred metabolites (the most abundant) have been reported as being reliably detected by NMR [168,257,258] using human urine, human CSF, and human serum metabolomes. While high-abundance metabolites are almost always physiologically important, low-concentration metabolites are often more important as diagnostic biomarkers. This means that NMR-based metabolomics are often unable to detect these important molecules or cannot be used in diagnostic clinical applications.

Overlapping resonances, especially for proton NMR spectra of biofluids, represent a continuing challenge in metabolite identification and quantification. Most NMR-based metabolomics studies are conducted using 1D ^1^H NMR spectroscopy, where the chemical shift dispersion is quite narrow, and most resonances are found in a small region between 1 to 4 ppm. This continues to cause significant challenges in peak assignment and compound identification, especially at lower magnetic field strengths. As mentioned above, 2D NMR experiments can be used to improve the reliability of peak assignments and reduce the spectral overlap problem. However, despite the continuous development of improved signal acquisition and processing methods in 2D NMR, a strong inertia still exists in the NMR community that limits its use in metabolomics studies. This likely is due to the continued (but not necessarily justifiable) concerns over data size and the relatively long acquisition times needed for 2D NMR. 

In general, NMR spectrometers are quite expensive compared to mass spectrometers or many other common analytical tools. Moreover, NMR instruments require highly skilled operators as well as substantial laboratory space with nonvibrational floors and isolation from magnetic and radio frequency interference. These factors, in addition to the overriding issue of low sensitivity, have made it challenging for NMR to expand its user base in metabolomics. In terms of translational clinical applications, the same issues regarding space, cost, and personnel also make it difficult for NMR to replace existing clinical analyzers for routine measurements in hospitals or clinics. As a general rule, mass spectrometry has had more success than NMR in the clinical chemistry lab because of its lower costs, its smaller instrumental footprint, and its ability to measure low-concentration metabolite species that are more frequently used in diagnostic assays.

## 8. Concluding Remarks and Future Prospects 

NMR spectroscopy plays important and multifaceted roles that have benefited and continue to benefit the field of metabolomics. These include biomarker discovery [51,259,260,261,262], kinetic and mechanistic metabolic studies [263,264,265,266,267], metabolite imaging [268,269,270], food and beverage characterization [271,272,273,274], environmental monitoring [275,276,277], and drug toxicity studies [278,279,280,281,282,283,284]. The unique characteristics of NMR, including its high level of reproducibility, its simplicity in sample preparation, its capacity to handle diverse sample types (liquids, solids, gels), its quantitative capabilities, and its utility in identifying unknown metabolites along with its nondestructive nature, have made it particularly useful in many diverse metabolomic disciplines. More recently, the advent of near total automation in the NMR workflow (including sample preparation, sample loading, spectral acquisition, and spectral deconvolution) means that NMR-based metabolomics is now becoming the preferred choice for many large-scale metabolomic studies. Near complete automation also opens the door to using NMR-based metabolomics in industrial, in clinical, in food production, and in food safety applications. 

While low sensitivity will continue to be viewed as an inherent limitation of NMR spectroscopy compared to mass spectrometry, recent improvements in probe design, magnet design, magnet field strength, pulse sequences, and methods for sensitivity enhancement are bringing NMR closer and closer to the sensitivity reported by many MS platforms. Indeed, the volume requirements of many quantitative MS-based metabolomic studies are now about the same as those needed for a 1 mm NMR probe (20–40 μL). Likewise, the lower limit of detection for ultra-high-field NMR instruments (high nanomolar concentrations) is not too different than what is seen for quantitative metabolomic studies performed on triple quadrupole MS instruments. What is not often acknowledged in MS-based metabolomics is that sample concentration and chemical derivatization are routinely used to boost sensitivity. Certainly, if the same protocols (of sample concentration and chemical derivatization with “enhancers”) were routinely used in NMR, it is possible that NMR experiments could claim sensitivity limits in the low-nanomolar level. 

As highlighted in the previous sections, there are a number of interesting new NMR technologies and new NMR techniques that are emerging that suggest NMR-based metabolomics will only get better. For instance, 1.2 GHz spectrometers will soon be available. These instruments should significantly increase the number of metabolites that can be detected by simple 1D ^1^H NMR spectroscopy. The appearance of liquid N_2_-cooled probes, while not as sensitive as their helium counterparts, should make cryoprobe costs substantially lower and, thereby, make cryoprobe use much more widespread in NMR laboratories. Low-cost DNP methods such as Sabre-sheath techniques [215,224,285,286] promise to make hyperpolarization sensitivity enhancement much more accessible and much more feasible for the average NMR lab. If routinely implemented, these hyperpolarization techniques could bring NMR sensitivity down to the low-nanomolar level. Fast acquisition methods represent another promising approach to improving NMR-based metabolomics, especially for well-studied biofluids such as serum or urine, where the location of the main spectral features and the composition of the biofluids are already well known. 

Standardization of NMR methods and data continues to be a challenge. Two “camps” in NMR-based metabolomics continue to exist with one camp tending to use statistical spectroscopy techniques that are combined with classical cheminformatics methods and the other camp tending to use spectral deconvolution techniques to identify and quantify metabolites. The lack of a common approach to NMR-based metabolomics combined with the difficulty in sharing or exchanging NMR data has limited some of the progress in this field. The recent introduction of nmrML as a common data exchange standard [287], the creation of metabolomic data deposition resources such as MetaboLights [288] and the Metabolomics WorkBench [289], along with the appearance of more freely available spectral deconvolution/identification software tools suggest that these problems with a lack of standardization or a lack of commonality may soon disappear. 

While some NMR spectroscopists have lamented the fact that NMR has been moved off its pedestal as one of the leading methods for structural biology or as the leading technology in metabolomics, we believe the future for NMR-based metabolomics is still very bright. Rather than viewing NMR as the only tool for metabolomics, it should be viewed as a vital, highly complementary tool to both LC-MS- and GC-MS-based metabolomics. It should also be noted that NMR still offers some unique advantages and fills some important holes that cannot be filled with other metabolomics technology platforms.

## Figures and Tables

**Table 1:** Summary of the most important advantages and limitations of nuclear magnetic resonance (NMR) spectroscopy compared to mass spectrometry (MS) in metabolomics applications.

	NMR	Mass Spectrometry
Reproducibility	High reproducibility is one of the fundamental advantages of NMR spectroscopy.	Compared to NMR spectroscopy, MS data are less reproducible.
Sensitivity	Intrinsically low but can be improved with multiple scans (time), higher magnet field strength, cryo-cooled and microprobes, and hyperpolarization methods.	High sensitivity is a major advantage of MS; metabolites with nanomolar concentrations can be readily detected
Selectivity	NMR is generally used for nonselective analysis. Peak overlaps from multiple detected metabolites pose major challenges.	MS is selective. However, in combination with chromatography (such as liquid and gas phase separation), it is a superior tool for targeted analysis.
Sample measurement	Enables relatively fast measurement using 1D ^1^H-NMR spectroscopy, where all metabolites at a detectable concentration level can be observed in one measurement.	Different ionization methods are required to maximize the number of detected metabolites.
Sample preparation	Involves minimal sample preparation, usually transferring the sample to an NMR tube and adding deuterated locking solvent. Can be automated.	More demanding; requires chromatography; requires sample derivatization for gas chromatography (GC)-MS.
Sample recovery	NMR is nondestructive and, hence, several analyses can be carried out on the same sample. Additionally, the sample can be recovered and stored for a long time.	MS is destructive technique; therefore, the sample cannot be recovered. However, it needs only a small amount of sample.
Quantitative analysis	NMR is inherently quantitative as the signal intensity is directly proportional to the metabolite concentrations and number of nuclei in the molecule.	The intensity of the MS line is often not correlated with metabolite concentrations as the ionization efficiency is also a determining factor.
Fluxomics Analysis	NMR permits both in vitro and in vivo metabolic flux analyses. Its inherently quantitative nature also enables precise quantification of precursors and products. Mapping of stable isotope locations and incorporating points in molecules is very easy via NMR.	MS can be used for fluxomics analysis; however, the destructive nature of MS-based methods means it is somewhat more limited than NMR-based fluxomics. In vivo fluxomics is not possible with MS, and isotope mapping is more difficult.
Tissue samples	Using high-resolution magic-angle sample spinning (HRMAS) NMR, it is possible to detect metabolites in tissue samples.	Although some MALDI-TOF approaches can be used to detect metabolites in tissue samples, these approaches are still far from being routine.
Number of detectable metabolites	Depending on spectral resolution, usually less than 200 metabolites can be unambiguously detected and identified in one measurement.	Using different MS techniques, it is possible to detect thousands of different metabolites and identify several hundred.
Targeted analysis	NMR spectroscopy can be used for both targeted and untargeted analyses, but it is not commonly used for targeted analyses.	Both GC-MS and liquid chromatography (LC)-MS are superior for targeted analyses
In vivo studies	Using magnetic resonance spectroscopy (MRS), in vivo investigation can be carried out most often using nuclei such as ^1^H and ^31^P.	Although desorption electrospray ionization (DESI) may be a useful way to analyze tissue samples during surgery, MS is not used for in vivo metabolomics studies.
